# Association between insulin receptor substrate-1 polymorphisms and high platelet reactivity with clopidogrel therapy in coronary artery disease patients with type 2 diabetes mellitus

**DOI:** 10.1186/s12933-016-0362-0

**Published:** 2016-03-22

**Authors:** Dingyu Zhang, Xiaolin Zhang, Dan Liu, Tengfei Liu, Wenzhi Cai, Chenghui Yan, Yaling Han

**Affiliations:** Cardiovascular Research Institute and Department of Cardiology, Shenyang Northern Hospital, 83 Wenhua Road, Shenyang, 110840 China

**Keywords:** Insulin receptor substrate-1, Single nucleotide polymorphism, High platelet reactivity, Clopidogrel, Coronary artery disease, Type 2 diabetes mellitus

## Abstract

**Background:**

The mechanisms leading to the high on-treatment platelet reactivity in diabetes patients are not fully elucidated. The genetic factors may be associated with the diminished antiplatelet efficacy of dual antiplatelet therapy. We investigated the possible association between insulin receptor substrate-1 (IRS-1) polymorphisms and high platelet reactivity in coronary artery disease (CAD) patients with type 2 diabetes mellitus (T2DM).

**Methods:**

A total of 674 CAD patients with T2DM were enrolled in this study. Platelet aggregation and platelet activation were assessed with light transmission aggregometry and flow cytometry analysis, respectively. Participants were divided into high platelet reactivity (HPR) group and non-HPR group according to their maximal platelet aggregation. Genotypes were identified by polymerase chain reaction and direct sequencing of genomic DNA. The association between IRS-1 genetic variants and platelet function was assessed.

**Results:**

There were 233 participants in the HPR group and 441 participants in the non-HPR group. G allele frequencies of rs13431554 were 27.7 % for the HPR group and 18.6 % for the non-HPR group (p < 0.001). Adenosine diphosphate and arachidonic acid induced platelet aggregation were significantly higher in G allele carriers compared with non-carriers (56.8 ± 16.2 vs 52.0 ± 17.9 %, p < 0.01, 28.9 ± 18.6 vs 25.2 ± 17.8 %, p < 0.01, respectively). We observed that P-selectin expression and PAC-1 binding were higher in G allele carriers compared with non-carriers (40.8 ± 12.4 vs 36.2 ± 13.8, p = 0.01; 43.7 ± 15.9 vs 38.7 ± 19.9, p = 0.03, respectively).

**Conclusion:**

The G allele of rs13431554 in the IRS-1 gene was associated with a hyperreactive platelet phenotype in the CAD patients with T2DM.

**Electronic supplementary material:**

The online version of this article (doi:10.1186/s12933-016-0362-0) contains supplementary material, which is available to authorized users.

## Background

Dual-antiplatelet therapy with aspirin and clopidogrel is the cornerstone of treatment for patients with cardiovascular disease [1]. Low response to antiplatelet therapy, characterized as high platelet reactivity (HPR), has been recognized as correlating with adverse events after acute coronary syndromes and percutaneous coronary intervention [2, 3]. The increased prevalence of low platelet inhibition following clopidogrel treatment and twofold to fourfold increased risk of cardiovascular disease were found in diabetes mellitus (DM) patients compared with non-DM patients [4–9]. Furthermore, platelet dysfunction in DM patients contributes to stent thrombosis and adverse events following percutaneous coronary intervention [10]. The mechanisms leading to the HPR of DM patients are not fully elucidated. Marin F, et al. demonstrated that single nucleotide polymorphisms (SNPs) were able to explain the variability in antiplatelet agents inefficacy [11]. Whether this may explain the heterogeneity of dual-antiplatelet therapy response in type 2 diabetes mellitus (T2DM) patients remains unexplored.

Several factors including up-regulation of platelet-signaling pathways have been involved in this interindividual response heterogeneity. Insulin inhibits platelet reactivity in the healthy individuals but not T2DM patients [4, 12]. T2DM patients have a loss of responsiveness to insulin that leads to increased platelet reactivity and reduced response to antiplatelet agents [11]. The loss of insulin signaling to Ca^2+^ regulating mechanisms in DM platelets was accompanied by abnormal signaling initiated by insulin receptor substrate-1 (IRS-1). IRS-1, as a ligand of insulin receptor tyrosine kinase, is central to the insulin signal transduction pathway [13]. Studies by Ferreira et al. suggested that insulin may cause platelet inhibition by activating IRS-1, which initiates the association with the Giα subunit coupled to the P2Y_12_ receptor [4]. Angiolillo et al. reported that the C allele of rs956115 in IRS-1 was associated with a hyperreactive platelet phenotype in Caucasian T2DM patients [14]. However, the associations are not reproduced across other ethnic populations. Whether there is other SNP in IRS-1 related to HPR in coronary artery disease (CAD) and T2DM patients remains to be elucidated.

The aim of this study was to investigate the relationship between the IRS-1 polymorphism and hyperreactive platelet phenotype in CAD patients with T2DM.

## Methods

### Study population

Patients with T2DM were eligible for the study if they were between 18 and 75 years, had undergone percutaneous coronary interventions (PCI), and been receiving standard dual antiplatelet therapy with aspirin (100 mg/day) and clopidogrel (75 mg/day) for at least 30 days. Patients treated with insulin and/or oral hypoglycemic medications for at least 1 month were enrolled. T2DM was defined according to the World Health Organization Report [15]. Exclusion criteria were as follows: a history of myocardial infarction fewer than 6 months prior to enrollment in the study; allergy/intolerance to aspirin or clopidogrel; impaired glucose tolerance or T2DM without pharmacological treatment, gestational diabetes, or transient hyperglycemia; use of oral anticoagulants, and antiplatelet agents other than aspirin and clopidogrel; recent treatment with a glycoprotein IIb/IIIa antagonist; using of proton pump inhibitors; end-stage renal or hepatic disease; treatment with fibrin-specific fibrinolytic therapy <24 h or non-fibrin-specific fibrinolytic therapy <48 h prior to randomization; presence of active internal bleeding or history of ischemic or hemorrhagic stroke in 6 months; platelet count <100 × 10^9^/L; hematocrit <25 %; creatinine levels >2.5 mg/dl; hepatic disease (hepatic enzymes twice the upper normal limit) Furthermore, to determine whether or not the findings were specific to patients with DM, a pharmacodynamics assessment was also extended to a cohort of CAD patients without DM. They also received standard dual antiplatelet therapy with aspirin and clopidogrel for at least 1 month after elective PCI with the stent implantation (Additional file 1: Figure S1).

This study confirmed to the ethical guidelines of the Helsinki declaration. The ethics approvals were obtained from Shenyang Northern Hospital ethics committee and all patients gave their informed written consents.

### Genotyping and haplotype analyses

Genomic DNA samples were extracted from peripheral blood lymphocytes of patients using TIANamp Blood DNA kits (Tiangen Biotech CO., LTD. Beijing, China). DNA concentration and quality were assessed using absorbance spectrophotometry and agarose gel electrophoresis, respectively. The selection of IRS-1 tag SNP was performed with GEVALT 2.0 software (GEnotype Visualization and Algorithmic Tool) [16, 17]. The SNP genotype data for China (CHB) population were downloaded from HapMap Project Browser, submitting a 100-kilobase pair region as a query, with a minor allele frequency cutoff of 0.05 and linkage disequilibrium (LD) measure r^2^ threshold of 0.8. LD blocks were determined according to the gerbil algorithm [16]. Haplotype frequency was determined by means of the algorithms implemented in the GEVALT software, using 0.05 as the frequency threshold to define common haplotypes. The specific primers were designed by primer 5.0 software (see Additional file 1: Table S1). Genotypes were identified by polymerase chain reaction (PCR) and direct sequencing of genomic DNA.

### Platelet function analysis

Platelet function analysis included assessments of platelet aggregation and platelet activation. Platelet aggregation was performed with light transmission aggregometry according to standard protocols [18–20]. Maximal platelet aggregation (MPA) was assessed with platelet-rich plasma (PRP) by the turbidimetric method using a four-channel Platelet Aggregation Chromogenic Kinetic System (Helena Laboratories,USA) after stimulus with adenosine diphosphate (ADP) or arachidonic acid (AA). PRP was prepared by centrifugation at 200*g* for 10 min. After adjustment from baseline, 20 μmol/L ADP or 1 mmol/L AA was added, and aggregation was recorded for 10 min. Results were expressed as a percentage of maximal light transmission from platelet-poor plasma obtained from the same patient. The cutoff value of HPR was defined as the upper quartile of MPA.

Platelet activation was determined by assessing platelet surface expression of activated glycoprotein (GP) IIb/IIIa and P-selectin as previously described [21]. A four-color flow cytometry (FACSCalibur, Becton–Dickinson BD, USA) was used for the assessment. 50 μl whole blood was stimulated in vitro with 5 μmol/L ADP before staining. The GPIIb/IIIa activation and P-selectin expression were assessed using fluorescein isothiocyanate-conjugated PAC-1 (PAC1-FITC) and phycoerythrin-conjugated anti-CD62P (CD62P-PE, Becton–Dickinson BD, USA). Whole blood was stained with an antibody mixture containing PAC1-FITC, CD62P-PE and PerCP peridinin chlorophyll protein-conjugated anti-CD61 (CD61-PerCP, Becton–Dickinson BD, USA) monoclonal antibodies and incubated for 20 min in the dark at room temperature. After incubation, 300 μL of 0.5 % PBS-buffered paraformaldehyde was added for fixation. Samples were analyzed within 2 h by flow cytometry. Platelet activation was expressed as the percentage of platelets positive for antibody binding.

### Statistical analysis

Continuous variables were presented as mean ± standard deviation. Categorical variables were presented as frequencies and percentages. They were used that Student’s t test for normally distributed variables and Mann–Whitney U test for non-normally distributed variables. χ^2^ test or Fisher exact test was used for categorical variables, as appropriate. Differences in allele and genotype frequencies between groups were analyzed using the χ^2^ test. P values were corrected for multiple comparisons for eight SNPs using the Bonferroni adjustment method, which changed the required significance level from <0.05 to <0.00625 (0.05 divided by eight). The χ^2^ test was used to determine whether individual polymorphisms were at Hardy–Weinberg equilibrium. Results with a two-tailed p value < 0.05 was considered statistically significant. Linkage disequilibrium (LD) analysis between each pair of SNP was assessed using GEVALT 2.0 software. The power of the sample size was calculated using QUANTO 1.2.4 software. Statistical analysis was performed using SPSS version 19.0 software.

## Results

### Baseline characteristics and platelet reactivity

Genotyping and platelet function analysis were performed in a total of 674 patients. Adenosine diphosphate (ADP)—induced maximum platelet aggregation was 53.8 ± 17.4 % for the 674 patients. The fourth quartile of maximum platelet aggregation was 65.8–86.5 %. HPR was defined as the 75th percentile of ADP-induced platelet aggregation (ADP-induced platelet aggregation >65.8 %). There were 233 patients with HPR and 441 patients without HPR. Baseline demographics, clinical characteristics, and laboratory data of the study populations were listed in Table 1. There were no significant differences between the HPR and non-HPR groups for all variables. In the DM population, AA-induced platelet aggregation was 44.3 ± 22.1 vs 22.9 ± 13.6 % in the HPR and non-HPR groups, respectively (p < 0.0001).

**Table 1:** Baseline demographic data and clinical characteristics of the study population

Variable	HPR (n = 233)	Non-HPR (n = 441)	*p*
Age, y	61.7 ± 9.3	62.0 ± 9.1	0.65
Males, n (%)	141 (60.5)	287 (65.1)	0.27
Smoking, n (%)	104 (44.6)	204 (46.3)	0.69
Hypertension, n (%)	169 (72.5)	292 (66.2)	0.09
BMI (kg/m^2^)	26.1 ± 3.4	25.9 ± 4.0	0.62
Triglycerides (mmol/L)	2.4 ± 1.8	2.3 ± 1.8	0.23
Total cholesterol (mmol/L)	4.2 ± 1.3	4.1 ± 1.1	0.12
LDL-C (mmol/L)	2.3 ± 0.9	2.2 ± 0.8	0.53
HDL-C (mmol/L)	1.1 ± 0.4	1.2 ± 0.4	0.85
HbA1C (%)	7.3 ± 1.1	7.4 ± 1.3	0.59
Hyperlipidemia, n (%)	127 (54.5)	223 (50.6)	0.33
Insulin-treated diabetes, n (%)	61 (26.2)	97 (22.0)	0.22
Previous MI, n (%)	48 (20.6)	102 (23.1)	0.45
Previous stroke, n (%)	27 (11.6)	36 (8.2)	0.15
Chronic renal dysfunction, n (%)	23 (9.9)	52 (11.8)	0.52
Essential medicine, n (%)			
ACEI/ARB	152 (62.5)	255 (57.8)	0.06
β-blocker	139 (59.7)	275 (62.4)	0.50
Calcium channel blocker	57 (24.5)	123 (27.9)	0.34
Statins	211 (90.6)	394 (89.3)	0.62
Nitrates	110 (47.2)	193 (43.8)	0.39

Data are expressed as mean ± SD, or n (%)

*BMI* body mass index; *LDL*-*C* low density lipoprotein cholesterol; *HDL*-*C* high density lipoprotein cholesterol; *HbA1C* hemoglobin A1C; *MI* myocardial infarction; *ACEI* angiotensin converting enzyme inhibitors; *ARB* angiotensin II receptor blockers

### Genotyping distribution of IRS1

We identified eight tag SNPs including one in the exon (rs1801278), one in the 3′ untranslated regions (3′ UTR) (rs13431554), three in the introns (rs2288586, rs1078533, 10205923) and three in the upstream and downstream regions(rs956115, rs1896832, rs2251692) of the IRS-1 gene. Information of the selected tagSNP was shown in Additional file 1: Table S2. Genotype distributions were all in Hardy–Weinberg equilibrium. The genotype and allele frequencies of the eight SNPs between the HPR and non-HPR groups were summarized in Table 2. Genotype distribution of rs13431554 was significantly different between the HPR and non-HPR groups (p < 0.001), which remained statistically significant after applying Bonferroni correction. The frequencies of AA, AG, and GG genotypes of rs13431554 in the HPR and non-HPR groups were 54.1, 36.5, 9.4, and 65.5, 31.7, 2.7 %, respectively. The G allele frequency of rs13431554 was significantly higher in the HPR group compared with the non-HPR group (27.7 vs 18.6 %, p < 0.001, odd ratio = 1.49, 95 % confidence interval = 1.22–1.82). There were no significant differences in allele frequencies or genotype distributions among the remaining seven SNPs between the HPR and non-HPR patients.

**Table 2:** Genotypes and allele frequencies of IRS-1 polymorphisms in primary participants

SNPs	Genotype/allele	HPR (n = 233)	Non-HPR (n = 441)	*p*	OR (95 % CI)
rs2251692	AA, no. (%)	100 (42.9)	193 (43.8)	0.24	
	AG, no. (%)	114 (48.9)	195 (44.2)		
	GG, no. (%)	19 (8.2)	53 (12.0)		
	A allele	314 (67.4)	581 (65.9)	0.58	0.96 (0.82–1.12)
	G allele	152 (32.6)	301 (34.1)		
rs13431554	AA, no. (%)	126 (54.1)	289 (65.5)	<0.001	
	AG, no. (%)	85 (36.5)	140 (31.7)		
	GG, no. (%)	22 (9.4)	12 (2.7)		
	A allele	337 (72.3)	718 (81.4)	<0.001	1.49 (1.22–1.82)
	G allele	129 (27.7)	164 (18.6)		
rs10205923	AA, no. (%)	28 (12.0)	39 (8.8)	0.24	
	AG, no. (%)	101 (43.2)	178 (40.4)		
	GG, no. (%)	105 (44.9)	224 (50.8)		
	A allele	157 (33.5)	256 (29.0)	0.09	1.16 (0.98–1.36)
	G allele	311 (66.5)	626 (71.0)		
rs1078533	AA no. (%)	5 (2.1)	9 (2.0)	0.68	
	AC no. (%)	40 (17.2)	88 (20.0)		
	CC no. (%)	188 (80.7)	344 (78.0)		
	A allele	50 (10.7)	106 (12.0)	0.48	0.88 (0.62–1.26)
	C allele	416 (89.3)	776 (88.0)		
rs2288586	CC, no. (%)	134 (57.5)	283 (64.2)	0.24	
	CG, no. (%)	87 (37.3)	139 (31.5)		
	GG, no. (%)	12 (5.2)	19 (4.3)		
	C allele	355 (76.2)	705 (79.9)	0.11	1.19 (0.96–1.46)
	G allele	111 (23.8)	177 (20.1)		
rs1801278	AA no. (%)	0 (0)	0 (0)	0.58	
	AG no. (%)	8 (3.4)	19 (4.3)		
	GG no. (%)	225 (96.6)	422 (95.7)		
	A allele	8 (1.7)	19 (2.2)	0.59	0.80 (0.35–1.81)
	G allele	458 (98.3)	863 (97.8)		
rs1896832	AA, no. (%)	165 (70.8)	284 (64.4)	0.20	
	AG, no. (%)	62 (26.6)	147 (33.3)		
	GG, no. (%)	6 (2.6)	10 (2.3)		
	A allele	392 (84.1)	715 (81.1)	0.16	0.84 (0.65–1.08)
	T allele	74 (15.9)	167 (18.9)		
rs956115	CC, no. (%)	2 (0.9)	10 (2.3)	0.28	
	CG, no. (%)	50 (21.5)	107 (24.3)		
	GG, no. (%)	181 (77.7)	324 (73.5)		
	C allele	54 (11.6)	137 (14.4)	0.15	1.03 (0.99–1.08)
	G allele	412 (88.4)	755 (85.6)		

In the cohort of CAD patients without DM, the frequencies of AA, AG and GG genotypes of rs13431554 in the HPR and non-HPR groups were 68.8, 22.9,8.3, and 63.6, 30.7, 5.7 % respectively, (p = 0.52). We found that there were no significant differences in genotype and allele frequencies between the HPR and non-HPR group in non-DM patients (Additional file 1: Table S3).

### Rs13431554 polymorphism and platelet activity

**Fig. 1:**
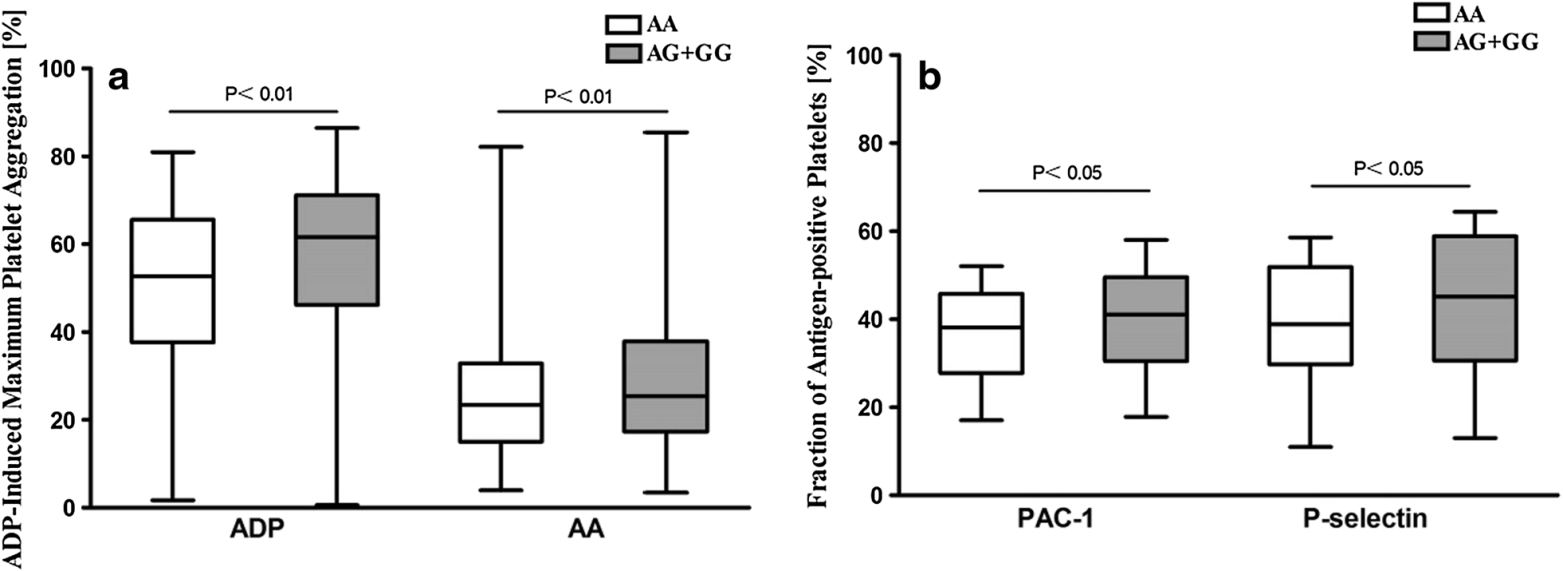
Platelet aggregation and platelet activation for rs13431554 genotypes. Platelet aggregation following adenosine diphosphate and arachidonic acid stumuli (**a**) and platelet activation (PAC-1 binding and P-selectin expression) following adenosine diphosphate stimuli (**b**) in the AA group compared with the AG + GG group according to rs13431554 genotype

Because of the low occurrence of patients with the GG genotype, the G allele carriers (AG + GG) were ascribed to a single group. Individuals who carried the G allele had higher platelet aggregation compared with non-carriers (Fig. 1a; Table 3) no matter with the ADP-or AA-induced platelet aggregation (56.8 ± 16.2 vs 52.0 ± 17.9 %, p < 0.01 for ADP; 28.9 ± 18.6 vs 25.2 ± 17.8 %, p < 0.01 for AA).

**Table 3:** Platelet function profiles according to rs13431554 genotype in study population

	DM patients		*p*	Non-DM patients		*p*
	AA	AG + GG		AA	AG + GG	
Platelet aggregation (%)						
ADP	52.0 ± 17.9	56.8 ± 16.2	<0.01	48.5 ± 17.4	51.1 ± 18.6	0.35
AA	25.2 ± 17.8	28.9 ± 18.6	<0.01	22.3 ± 14.9	24.8 ± 16.4	0.29
Platelet activation (%)						
PAC-1	36.2 ± 13.8	40.8 ± 12.4	0.01	34.8 ± 11.8	36.5 ± 13.1	0.37
P-selectin	38.7 ± 19.9	43.7 ± 15.9	0.03	36.4 ± 17.0	39.0 ± 16.2	0.31

Data are expressed as mean ± SD of percentages of platelet aggregation or percentages of positive platelets

*ADP* adenosine diphosphate; *AA* arachidonic acid

Platelet surface expression of activated glycoprotein IIb/IIIa and P-selectin were assessed to confirm the association between platelet activation and the alleles of rs13431554. P-selectin expression and glycoprotein IIb/IIIa activation on the platelet surface were increased in the rs13431554 G allele carriers (40.8 ± 12.4 vs 36.2 ± 13.8 %, p = 0.01 for PAC-1; 43.7 ± 15.9 vs 38.7 ± 19.9 %, p = 0.03 for P-selectin) (Fig. 1b; Table 3).

In the external validation cohort of patients without DM, the ADP-induced platelet aggregations were 48.5 ± 17.4 and 51.1 ± 18.6 % (p = 0.35), and AA-induced platelet aggregations were 22.3 ± 14.9 and 24.8 ± 16.4 % (p = 0.29) between G allele carriers and non-carriers. There was no significant difference between the rs13431554 G genotype and platelet activation in non-DM patients (34.8 ± 11.8 vs 36.5 ± 13.1 %, p = 0.37 for PAC-1; 36.4 ± 17.0 vs 39.0 ± 16.2 %, p = 0.31 for P-selectin) (Table 3).

### IRS-1 haplotypes analysis

**Fig. 2:**
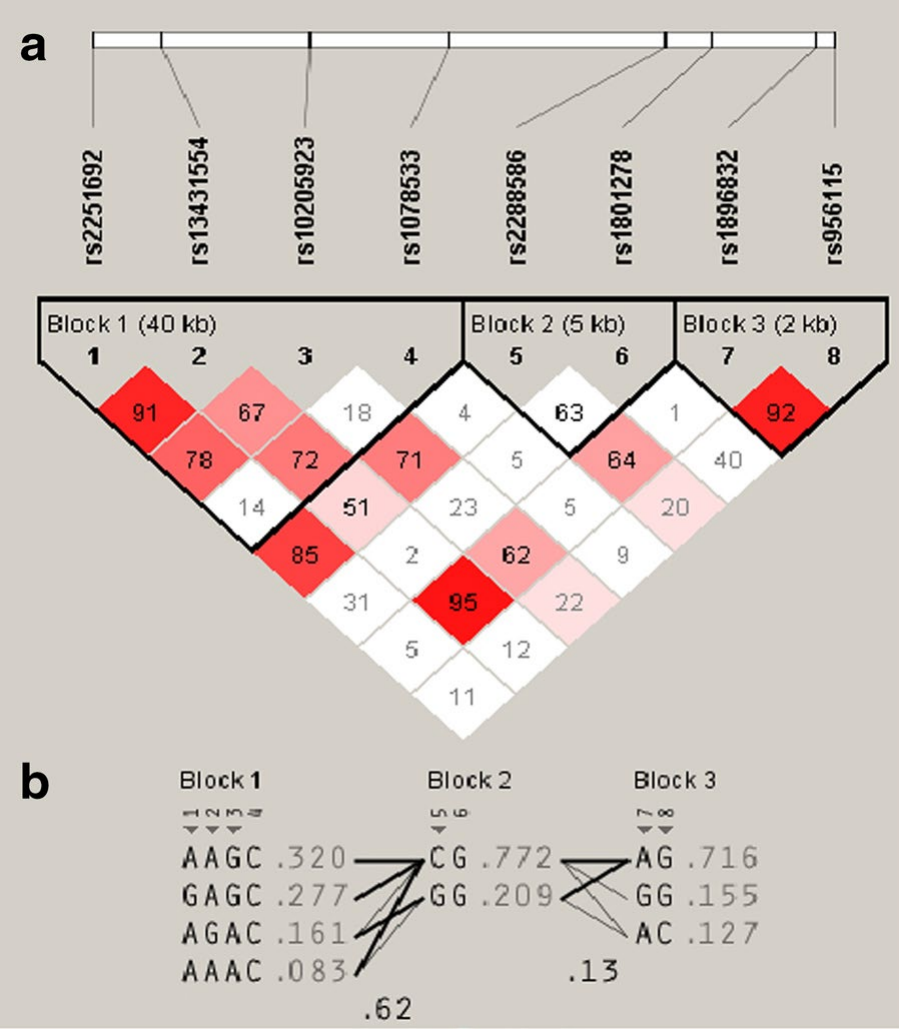
Linkage disequilibrium *plot* for SNPs in the IRS-1 gene. Linkage disequilibrium between markers was measured by pairwise D’ as calculated from the sample of individuals studied. **a** Linkage disequilibrium *blocks* were identified as suggested by the gerbil algorithm. *Color* support for D’ estimation as reported in the computer program *Haploview* (Barrett JC et al., 2005). *White* D’ <1 and LOD <2; *shades* of *pink/red* D’ <1 and LOD ≥2; *red* D’ = 1 and LOD ≥2. Number in the *square*: D’ value expressed as estimated D’ value × 100; *empty square* D’ = 1. **b** Haplotypes with a frequency >5 % in the two linkage disequilibrium *blocks*

LD analysis of the eight SNPs revealed three LD blocks, as defined according to the gerbil algorithm (see Additional file 1: Table S4). Three haplotype LD blocks were identified for the IRS-1 gene region: block-1 (rs2251692-rs13431554-rs10205923-rs1078533); block-2 (rs2288586-rs1801278); and block-3 (rs1896832-rs956115) (Fig. 2a). Haplotype frequencies were shown in Fig. 2b.

We investigated the associations between haplotype LD block frequencies and high platelet activity. There was no significant association among the three blocks with any of the phenotypes investigated, even though haplotype block-1 (AGAC) frequency exactly corresponded to the G allele of rs13431554.

## Discussion

In healthy subjects, platelets are inhibited by insulin leading to reduced Ca^2+^ mobilization and aggregate formation [8]. Studies have demonstrated that platelet hyperactivity in T2DM is likely to be caused by a defect in the mechanisms through which insulin interferes with signaling by the P2Y_12_ receptor. After binding with its receptor, insulin activates IRS-1 through tyrosine phosphorylation, which initiates the association with the Giα-subunit. Then, Giα activity is inhibited and impairs suppression of adenylyl cyclase through P2Y_12_. Patients with T2DM are at particularly risk for restenosis and repeat the revascularization procedures because of the increased platelet activation and the reduced platelet inhibition by antiplatelet agents [22–25].

This study suggested that IRS-1 polymorphism has an effect on platelet response to dual-antiplatelet therapy in CAD patients with T2DM. The eight SNPs of IRS-1 gene were examined to study the association between the polymorphisms and the hyperreactive platelet phenotype. We found that the G allele of IRS-1 rs13431554 polymorphism was significant associated with high platelet activity. The variant is located in the 3′UTR region of IRS-1, which is more important for mRNA stability, localization, and translational efficiency.

It has been reported that the binding affinity between miRNA and its target mRNA may be changed by SNP located at miRNA target sites [26]. SNP variant may lead to the degradation of mRNA and the inhibition of mRNA translation into protein [27]. Based on our bioinformatics analysis using the microRNA.org database [28], has-miR-23 and has-miR-130 which binding to the sequence surrounding the variant site were identified. These bioinformatics forecasts indicated that rs13431554 may affect the binding affinity between IRS-1 mRNA and miRNAs, which may alter the expression of IRS-1 gene. It is reasonable to suggest that rs13431554 might influence miRNA biogenesis and function, also contribute to platelet activity. Further functional evaluations are needed to confirm this hypothesis.

The heterogeneity of antiplatelet drug efficacy is known to vary according to the genetic background. Cytochrome P450 (CYP) polymorphism correlates with the diminished antiplatelet efficacy of clopidogrel and the high risk for adverse cardiovascular events following stent implantation [29–32]. The impact of CYP2C19 polymorphism on platelet reactivity to clopidogrel seems to be more significant in non-DM patients compared with DM patients [33]. Precise clinical phenotypes and functional characterizations of gene variants are currently lacking. An early study reported that T2DM patients with the C allele of the rs956115 marker of the IRS-1 gene have a hyperreactive platelet phenotype and increased risk of major adverse cardiac events [14]. However, this result was not observed in our study. Possible explanation for these divergent results may lie in the ethnic differences in genetic backgrounds. The rs956115 polymorphism is located in the 5′region of the IRS-1 gene which doesn’t affect amino acid coding and protein function. The rs13431554 polymorphism is located in in the 3′region of the IRS-1 gene, and may be more important to the function of IRS-1. We found that the outcomes were special to DM patients because there was no significant association between IRS-1 polymorphism and platelet activity in non-DM cohort. We did not find the association between the haplotype LD blocks and high platelet activity phenotypes in our study.

Glycemic control has an effect on platelet reactivity in diabetic patients. Previous study showed that a reduction in HbA1c level was associated with a reduction in blood thrombogenicity. Grzegorz Gajos et al. demonstrated that fasting glycemia <4.5 mmol/L was associated with enhanced thrombin formation in T2DM patients, especially when strict glycemic control was achieved (HbA1c <6.0 %) [34]. Patients in our study had got a good glycemic control and limited variability in HbA1C levels, so the level of HbA1C didn’t affect our study results.

More strategies are being studied to overcome increased platelet reactivity in DM patients. Previous study showed that using a loading dose of clopidogrel rather than small daily doses was not sufficient for adequately overcoming increased platelet reactivity in DM patients, highlighting the need for more effective anti-platelet drugs for such patients [35]. And, novel P2Y12 receptor blockers such as prasugrel and ticagrelor were found that they were superiors in terms of pharmacodynamics and pharmacokinetic effects than clopidogrel [36–38]. Recent randomized studies also demonstrated that ticagrelor was superior to prasugrel for reducing platelet reactivity in subjects with diabetes [39].

This is the first study to evaluate the association between IRS-1 polymorphism and high on-treatment platelet reactivity in a population of Chinese CAD patients with DM. Identification of susceptible genes that contribute to the declining platelet response may facilitate prediction, prevention, and development of improved antiplatelet treatments.

### Study limitations

There is a lack of standardization identification of optimal cutoff values for defining the HPR [40]. Standardized definitions of defining individual responsiveness to clopidogrel should be used in further experiments. We assessed platelet reactivity in vitro, that can’t fully represent platelet phenotype in circulating condition. The mechanisms of the rs13431554 variant contributing to the poor response to clopidogrel in T2DM patients remain speculative. Further functional evaluations with larger sample sizes are needed to confirm our findings. Different geographical and ethnic backgrounds of the study participants may affect the results of any association study. Therefore, more patients of different geographical areas and ethnicities should be included to confirm our conclusions in the further studies.

## Conclusions

Variant rs13431554 in the IRS-1 gene is associated with a hyperreactive platelet phenotype and a sub-optimal response to antiplatelet drug in CAD patients with T2DM. There is an association between the G allele of rs13431554 and the increased platelet aggregation and activity. Further validations using larger sample sizes of diverse ethnic populations and functional evaluations are warranted.

## Additional file

10.1186/s12933-016-0362-0 Flow diagram of the study cohorts. **Table S1.** Primers, PCR product lengths, and reaction conditions of selected tagSNPs. **Table S2.** Information on genotyped SNPs of IRS-1. **Table S3.** Genotypes and allele frequencies of IRS-1 polymorphisms according to platelet activity in non-DM patients. **Table S4.** Linkage disequilibrium measurements between SNP pairs of eight tag SNPs of the IRS-1 gene. **Table S5.** Genotypes and allele frequencies of IRS-1 polymorphisms in DM and non-DM patients. **Table S6.** Baseline demographic data and clinical characteristics according to the rs13431554.

## Abbreviations

HPR: high platelet reactivity
DM: diabetes mellitus
T2DM: type 2 diabetes mellitus
IRS-1: insulin receptor substrate-1
SNP: single nucleotide polymorphisms
CAD: coronary artery disease
PCI: percutaneous coronary interventions
LD: linkage disequilibrium
MPA: maximal platelet aggregation
PRP: platelet-rich plasma
ADP: adenosine diphosphate
AA: arachidonic acid
