# Supplementary material for: Association between insulin receptor substrate-1 polymorphisms and high platelet reactivity with clopidogrel therapy in coronary artery disease patients with type 2 diabetes mellitus
Source: Cardiovasc Diabetol. 2016 Mar 22;15:50. doi: 10.1186/s12933-016-0362-0 (PMC4804508; doi:10.1186/s12933-016-0362-0)
Supplement: Supplementary file 1 — 10.1186/s12933-016-0362-0 Flow diagram of the study cohorts. Table S1. Primers, PCR product lengths, and reaction conditions of selected tagSNPs. Table S2. Information on genotyped SNPs of IRS-1. Table S3. Genotypes and allele frequencies of IRS-1 polymorphisms according to platelet activity in non-DM patients. Table S4. Linkage disequilibrium measurements between SNP pairs of eight tag SNPs of the IRS-1 gene. Table S5. Genotypes and allele frequencies of IRS-1 polymorphisms in DM and non-DM patients. Table S6. Baseline demographic data and clinical characteristics according to the rs13431554. [file 12933_2016_362_MOESM1_ESM.docx]

**Supplemental Materials**

**Association between insulin receptor substrate-1 polymorphisms and high platelet reactivity with clopidogrel therapy in coronary artery disease patients with type 2 diabetes mellitus**

Dingyu Zhang, Xiaolin Zhang, Dan Liu, Tengfei Liu, Wenzhi Cai, Chenghui Yan, Yaling Han*.

Cardiovascular Research Institute and Department of Cardiology, Shenyang Northern Hospital, 83 Wenhua Road, Shenyang 110840, China

87 patients were excluded for meeting exclusion criteria

Test platelet aggregation and genotyping (n=213)

CAD patients without T2DM on standard aspirin plus clopidogrel therapy for at least 30 days (n=300)

16 patients were excluded for failing to extract DNA

9 patients were excluded for failing genotyping

Finally 188 eligible patients were enrolled in this study

147 patients were excluded for meeting exclusion criteria

122 patients were excluded for not receiving hypoglycemic therapy

Test platelet aggregation and genotyping (n=705)

CAD patients with T2DM on standard aspirin plus clopidogrel therapy for at least 30 days (n=974)

11 patients were excluded for failing to extract DNA

17 patients were excluded for failing genotyping

Finally 674 eligible patients were enrolled in this study

**Figure S1.** Flow diagram of the study cohorts

**Table S1.** Primers, PCR product lengths, and reaction conditions of selected tagSNPs

| **SNPs** | **Primers** | **length (bp)** | **PCR reaction conditions** |
| --- | --- | --- | --- |
| rs2251692 | F:5’-GCCACCATTAGCAATCAG-3’ | 476 | 94℃4m(94℃30s-57℃30s-72℃40s)×30-72℃7m-4℃∞ |
|  | R:5’-ACAGGTTCAAGCAGGAGAC-3’ |  |  |
| rs13431554 | F:5’-AGGGTTTCACCATCCACT-3’ | 317 | 94℃4m(94℃30s-55℃30s-72℃40s)×30-72℃7m-4℃∞ |
|  | R:5’-TTACCTCCAACTAACATTC-3’ |  |  |
| rs10205923 | F:5’-GGTGGGAGGATTGCTAAG-3’ | 453 | 94℃4m(94℃30s-57℃30s-72℃40s)×30-72℃7m-4℃∞ |
|  | R:5’-CAATCAAGGAAACCCTCA-3’ |  |  |
| rs1078533 | F:5’-CACATTTCTCCTCTGCCA-3’ | 309 | 94℃4m(94℃30s-57℃30s-72℃40s)×30-72℃7m-4℃∞ |
|  | R:5’-GTCGAAGTTGAAACGTGAA-3’ |  |  |
| rs2288586 | F:5’-TGACAAGGGTTTGGGTAG-3’ | 445 | 94℃4m(94℃30s-58℃30s-72℃40s)×30-72℃7m-4℃∞ |
|  | R:5’-CAGTAGCAGTTTTGGGTCA-3’ |  |  |
| rs1801278 | F:5’-GTCTGGCTACTTGTCTGGC-3’ | 273 | 94℃4m(94℃30s-57℃30s-72℃40s)×30-72℃7m-4℃∞ |
|  | R:5’-ACAACTCATCTGCATGGTCA-3’ |  |  |
| rs1896832 | F:5’-CTCTGTTACCCAGGCTAGAG-3’ | 335 | 94℃4m(94℃30s-57℃30s-72℃40s)×30-72℃7m-4℃∞ |
|  | R:5’-GAAGAAGCACGCTTAGTGAA-3’ |  |  |
| rs956115 | F:5’-ATAGAGATTCCAAGAGTTATG-3’ | 365 | 94℃4m(94℃30s-56℃30s-72℃40s)×30-72℃7m-4℃∞ |
|  | R:5’-ATTGCCAAGATATGTCCTAA-3’ |  |  |

F:forward primer; R:reverse primer; bp:[base pairs](app:ds:base-pairs); m:minute; s:second

**Table S2.** Information on genotyped SNPs of IRS-1

| **SNP** | **Position** | **Region in Gene** | **MAF** | **HW p-values** |
| --- | --- | --- | --- | --- |
| rs2251692 | 226725064 | 3' Flanking | 0.34 | 0.48 |
| rs13431554 | 226732872 | 3' UTR | 0.22 | 0.68 |
| rs10205923 | 226749841 | Intron | 0.31 | 0.49 |
| rs1078533 | 226765871 | Intron | 0.12 | 0.06 |
| rs2288586 | 226790674 | Intron | 0.21 | 0.96 |
| rs1801278 | 226795828 | Exon | 0.02 | 0.60 |
| rs1896832 | 226807770 | 5' Flanking | 0.18 | 0.17 |
| rs956115 | 226809848 | 5' Flanking | 0.13 | 0.96 |

MAF: minor allele frequency

**Table S3.** Genotypes and allele frequencies of IRS-1 polymorphisms according to platelet activity in non-DM patients

|  | **HPR** | **Non-HPR** |  |  |
| --- | --- | --- | --- | --- |
| **Genotype/allele** | **(n=48)** | **(n=140)** | ***p*** | **OR (95% CI)** |
| AA , no. (%) | 33(68.8) | 89(63.6) | 0.52 |  |
| AG, no. (%) | 11(22.9) | 43(30.7) |  |  |
| GG, no. (%) | 4(8.3) | 8(5.7) |  |  |
| A allele | 77(80.2) | 221(78.9) | 0.88 | 0.94(0.59-1.49) |
| G allele | 19(19.8) | 59(21.1) |  |  |

**Table S4.** Linkage disequilibrium measurements between SNP pairs of eight tag SNPs of the IRS-1 gene

| SNP1 | SNP2 | D' | LOD | r^2^ | 95%CI |
| --- | --- | --- | --- | --- | --- |
| rs2251692 | rs13431554 | 0.916 | 21.72 | 0.112 | 0.81-0.97 |
| rs2251692 | rs10205923 | 0.78 | 22.86 | 0.132 | 0.67-0.86 |
| rs2251692 | rs1078533 | 0.142 | 0.65 | 0.005 | 0.02-0.29 |
| rs2251692 | rs2288586 | 0.853 | 17.97 | 0.097 | 0.73-0.93 |
| rs2251692 | rs1801278 | 0.31 | 0.15 | 0.001 | 0.03-0.73 |
| rs2251692 | rs1896832 | 0.05 | 0.14 | 0.001 | -0.01-0.18 |
| rs2251692 | rs956115 | 0.119 | 0.6 | 0.004 | 0.01-0.25 |
| rs13431554 | rs10205923 | 0.677 | 38.96 | 0.282 | 0.59-0.75 |
| rs13431554 | rs1078533 | 0.728 | 3.05 | 0.016 | 0.41-0.88 |
| rs13431554 | rs2288586 | 0.519 | 33.99 | 0.269 | 0.44-0.59 |
| rs13431554 | rs1801278 | 0.026 | 0.01 | 0.0 | 0.0-0.36 |
| rs13431554 | rs1896832 | 0.955 | 9.97 | 0.045 | 0.79-0.99 |
| rs13431554 | rs956115 | 0.127 | 1.21 | 0.009 | 0.03-0.24 |
| rs10205923 | rs1078533 | 0.182 | 1.22 | 0.009 | 0.05-0.32 |
| rs10205923 | rs2288586 | 0.718 | 43.97 | 0.317 | 0.63-0.79 |
| rs10205923 | rs1801278 | 0.235 | 0.07 | 0.0 | 0.02-0.71 |
| rs10205923 | rs1896832 | 0.625 | 5.36 | 0.031 | 0.42-0.76 |
| rs10205923 | rs956115 | 0.226 | 2.41 | 0.017 | 0.1-0.35 |
| rs1078533 | rs2288586 | 0.048 | 0.01 | 0.0 | 0.0-0.41 |
| rs1078533 | rs1801278 | 0.056 | 0.07 | 0.001 | 0.0-0.33 |
| rs1078533 | rs1896832 | 0.051 | 0.23 | 0.002 | -0.01-0.16 |
| rs1078533 | rs956115 | 0.098 | 0.97 | 0.008 | 0.02-0.2 |
| rs2288586 | rs1801278 | 0.634 | 0.38 | 0.002 | 0.07-0.9 |
| rs2288586 | rs1896832 | 0.641 | 3.59 | 0.02 | 0.38-0.8 |
| rs2288586 | rs956115 | 0.2 | 2.89 | 0.022 | 0.1-0.31 |
| rs1801278 | rs1896832 | 0.019 | 0.0 | 0.0 | 0.01-0.74 |
| rs1801278 | rs956115 | 0.402 | 0.08 | 0.0 | 0.03-0.86 |
| rs1896832 | rs956115 | 0.927 | 5.23 | 0.024 | 0.66-0.98 |

CI: low and high boundaries of 95% confidence intervals for D' value;

D': Lewontin's normalized measure of allelic association, ranging from 0 (no association) to 1 (association);

r^2^: correlation coefficient of marker pairs;

LOD: log of odds for allelic association (values≥2 are considered to support evidence of allelic

association).

**Table S5.** Genotypes and allele frequencies of IRS-1 polymorphisms in DM and non-DM patients

|  | **DM** | **Non-DM** |  |  |
| --- | --- | --- | --- | --- |
| **Genotype/allele** | **(n=674)** | **(n=188)** | ***p*** | **OR (95% CI)** |
| AA , no. (%) | 415(61.6) | 122(64.9) | 0.42 |  |
| AG, no. (%) | 225(33.4) | 54(28.7) |  |  |
| GG, no. (%) | 34(5.0) | 12(6.4) |  |  |
| A allele | 1055(78.3) | 298(79.3) | 0.72 | 1.05(0.84-1.31) |
| G allele | 293(21.7) | 78(20.7) |  |  |

**Table S6.** Baseline demographic data and clinical characteristics according to the rs13431554 genotypes

| **Variable** | **AA**  **(n=415)** | **AG+GG**  **(n=259)** | ***p*** |
| --- | --- | --- | --- |
| Age, y | 62.3±9.6 | 61.5±8.3 | 0.23 |
| Males, n (%) | 271(64.3) | 157(60.6) | 0.25 |
| Smoking, n (%) | 199(48.0) | 109(42.1) | 0.15 |
| Hypertension, n (%) | 281(67.7) | 180(69.5) | 0.67 |
| BMI(kg/m^2^) | 26.0±3.8 | 26.0±4.3 | 0.83 |
| Triglycerides (mmol/L) | 2.4±1.6 | 2.2±1.6 | 0.29 |
| Total cholesterol (mmol/L) | 4.2±1.2 | 4.0±1.2 | 0.13 |
| LDL-C (mmol/L) | 2.3±0.9 | 2.2±0.9 | 0.19 |
| HDL-C (mmol/L) | 1.2±0.4 | 1.1±0.5 | 0.38 |
| HbA1C (%) | 7.4±1.1 | 7.3±1.4 | 0.63 |
| Hyperlipidemia, n (%) | 214(51.6) | 136(52.5) | 0.81 |
| Insulin-treated diabetes, n (%) | 93(22.4) | 65(25.1) | 0.46 |
| Previous MI, n (%) | 99(23.9) | 51(19.7) | 0.22 |
| Previous stroke, n (%) | 44(10.6) | 19(7.3) | 0.18 |
| Chronic renal dysfunction, n (%) | 37(8.9) | 18(6.9) | 0.39 |
| Essential medicine |  |  |  |
| ACEI/ARB, n (%) | 247(59.5) | 160(61.8) | 0.57 |
| β-blocker, n (%) | 250(60.2) | 164(63.3) | 0.46 |
| Calcium channel blocker, n (%) | 117(28.2) | 63(24.3) | 0.28 |
| Statins,n(%) | 373(89.9) | 232(89.6) | 0.90 |
| Nitrates, n (%) | 182(43.9) | 121(46.7) | 0.48 |

Data are expressed as mean ± SD, or n (%); ACEI: angiotensin converting enzyme inhibitors; ARB: angiotensin II receptor blockers; BMI: body mass index; MI: myocardial infarction; LDL-C: low density lipoprotein cholesterol; HDL-C: high density lipoprotein cholesterol; HbA1C: hemoglobin A1C
